# Hypothesis paper: high prevalence of Tinel sign in hypermobile Ehlers-Danlos syndrome

**DOI:** 10.3389/fneur.2025.1508176

**Published:** 2025-10-31

**Authors:** Shahreyar Shar Hashemi, Dacre R. T. Knight

**Affiliations:** ^1^Nerve Bone & Joint Institute, Washington, DC, United States; ^2^Department of Medicine, University of Virginia, Charlottesville, VA, United States

**Keywords:** Tinel sign, hypermobile Ehlers-Danlos syndromes, cubital tunnel syndrome, carpal tunnel syndrome, neuralgia, neuropathy

## Abstract

Ehlers-Danlos syndromes (EDS) encompass a group of genetic connective tissue disorders that affect the structure and function of proteins and enzymes that provide stability to the body. The hypermobile subtype of EDS (hEDS) is the most common and is characterized by joint hypermobility, skin hyperextensibility, and widespread musculoskeletal pain. Neuropathic symptoms, including pain, numbness, tingling, and weakness, are increasingly recognized in this population. This paper proposes a study to test the hypothesis that hEDS individuals will have a higher prevalence of positive Tinel signs compared to controls, indicating heightened sensitivity to nerve irritation or compression in hEDS that can guide better diagnosis and treatment.

## Introduction

Ehlers-Danlos syndromes (EDS) are a group of inherited connective tissue disorders primarily affecting the structure and function of collagen and related proteins (1, 2). Among these, the hypermobile subtype (hEDS) is the most prevalent and is characterized by joint hypermobility, skin hyperextensibility, and chronic musculoskeletal pain (2). In addition to these hallmark features, individuals with hEDS frequently report neuropathic symptoms such as pain, numbness, tingling, and weakness (3).

The structural fragility inherent in hEDS, resulting from collagen dysfunction, may predispose individuals to peripheral nerve irritation or entrapment. Connective tissue laxity contributes to joint instability and recurrent subluxations, potentially increasing mechanical stress on adjacent nerves (4). Although each subtype of EDS, with the exception of hEDS, has an identified molecular basis, all share features that may contribute to increased nerve vulnerability (1, 2). These biomechanical factors suggest a plausible link between hEDS and heightened susceptibility to nerve-related symptoms.

Tinel's sign—a clinical marker characterized by tingling sensations elicited by percussion over a nerve—is commonly used to assess nerve irritation or regeneration (5, 6). It is frequently positive in nerve entrapment syndromes such as carpal tunnel and cubital tunnel syndromes. Given the propensity for joint instability and mechanical strain in hEDS, it is hypothesized that individuals with this condition will demonstrate a higher prevalence of positive Tinel signs, reflecting increased nerve sensitivity or chronic irritation.

Prior studies have documented an association between EDS and peripheral neuropathies, including entrapment syndromes like thoracic outlet syndrome, carpal tunnel syndrome, and others (1–4). However, no systematic investigation has evaluated the prevalence of Tinel's sign in the hEDS population. This study seeks to address this gap by assessing the frequency and distribution of positive Tinel signs in individuals with hEDS, thereby contributing to a deeper understanding of the neuropathic manifestations associated with this condition.

## Hypothesis

Individuals with hypermobile Ehlers-Danlos syndrome (hEDS) will exhibit a significantly higher prevalence of positive Tinel's signs compared to the general population due to increased susceptibility to nerve irritation and compression.

### Proposed mechanism

The underlying mechanism of Tinel signs in hEDS individuals is possibly multifactorial. This may include increased joint laxity and subluxation as frequent joint instability and subluxation may subject peripheral nerves to increased mechanical stress, leading to irritation and subsequent positive Tinel's signs. Previous reports have raised concern for brachial plexopathies playing a role in the pathogenesis of neuropathy in patients with EDS type III (hEDS), in the absence of outright trauma (7). Microtrauma to nerves: in hEDS individuals, the lack of sufficient support from connective tissues may allow minor, repetitive trauma to nerves, causing micro-injuries that manifest as nerve hypersensitivity. Inflammatory response and neuropathic changes: chronic mechanical stress on nerves may trigger local inflammation, further exacerbating nerve irritation and contributing to a positive Tinel's response. While the direct pathophysiology is still unclear, previous retrospective and prospective research has shown that nerve fiber loss and neuropathy—evidenced by biopsy and sensory testing—occurs more often in patients with hEDS compared to controls (8, 9). Increased vulnerability of the nerves to stress and compression: joint instability may predispose to malalignment of the nerve pathway leading compression or focal neuropathic lesion. This has been revealed in similar scenarios and case reports of postural causes of neuropathy (10).

### Proposed study

A prospective cross-sectional study is proposed for the study design. Two groups will be assessed—a cohort of individuals diagnosed with hEDS and a control group from the general population with no history of connective tissue disorders or joint hypermobility. The inclusion criteria for the hEDS group will consist of individuals meeting the 2017 International Classification criteria for hypermobile EDS (1). The control group will be matched for age and sex. Small fiber neuropathy individuals will be in a separate subgroup. The exclusion criteria will be the presence of confounding causes of neuropathy, such as thyroid disease, amputation, and stroke.

Regarding the assessment for Tinel sign, the presence of a Tinel sign will be determined on physical exam as previously described in the literature (11). In the lower extremity, with palpation at the anatomic entrapment site, tingling, shock-like, or electrical sensations radiating along the nerve's distribution will be equivalent to a positive Tinel sign. For example, a positive Tinel Sign will be noted if the patient experiences pain and tingling with palpation at the fibular neck (i.e., common peroneal nerve compression). The presence of neuropathy will be determined by the score of the Michigan Neuropathy Symptom Index patient version test, with the presence of neuropathy being defined as a score of 4 or greater (12). Tinel sign will be assessed bilaterally at multiple common sites of nerve compression, sensory, and motor neurological examination on the innervation field of each nerve including but not limited to locations in Table 1.

**Table 1:** Tinel signs criteria by tunnel syndrome.

**Nerve**	**Tunnel/Region**	**Percussion site**	**Positive response**
Brachial plexus	Supraclavicular	Above clavicle, lateral to neck	Tingling in entire arm or C5–T1 dermatomes
Axillary nerve	Quadrangular space	Posterior shoulder, below glenohumeral joint	Tingling in deltoid and lateral upper arm
Dorsal scapular nerve	Scapular tunnel	Medial scapular border	Deep aching or tingling in scapular region
Radial nerve	Radial tunnel	4–5 cm distal to lateral epicondyle	Tingling in dorsal forearm or radial hand
Median nerve	Proximal forearm (Pronator)	Between heads of pronator teres	Tingling in thumb, index, middle, 1/2 ring finger
Median nerve	Carpal tunnel	Volar wrist, over flexor retinaculum	Same as above
Ulnar nerve	Cubital tunnel	Posterior to medial epicondyle	Tingling in ring and little fingers, ulnar forearm
Sciatic nerve	Piriformis	Midpoint between greater trochanter & ischial tuberosity	Tingling down posterior thigh/leg/foot
Femoral nerve	Inguinal tunnel	Below inguinal ligament, lateral to femoral artery	Tingling in anterior thigh, medial leg
Lateral femoral cutaneous	ASIS tunnel	Just inferior/medial to ASIS	Tingling in anterolateral thigh
Common peroneal nerve	Fibular tunnel	At fibular neck	Tingling in lateral leg and dorsum of foot
Superficial peroneal nerve	Lower leg tunnel	Lateral mid-leg	Tingling in anterolateral leg, dorsum of foot
Deep peroneal nerve	Ankle tunnel	Lateral to EHL tendon at ankle	Tingling in first web space
Tibial nerve	Popliteal tunnel	Midline of popliteal fossa	Tingling in plantar foot, posterior calf
Posterior tibial nerve	Tarsal tunnel	Posterior to medial malleolus	Tingling in sole of foot and toes

Sensory and motor nerve conduction velocity (NCV) studies across the potential entrapment sites above will be completed on at least four selected nerves which can most easily be examined, to include: median nerve at the wrist, ulnar nerve at the elbow, fibular nerve at the fibular head segment and tibial nerve at the ankle. NCV testing will be performed with surface stimulation and recording. An electrode pair will be put over the skin on the given peripheral nerve site to apply supramaximal stimulation. Recording electrodes will be placed over the muscles innervated by the given nerve (motor NCV), as well as those recorded over the nerve trunks (sensory or mixed NCV) (13). Stimulation and recording points for each nerve to be examined are listed in Table 2.

**Table 2:** Electrode placement and recording points for nerve conduction studies.

**Nerve**	**Stimulation site(s)**	**Electrode placement (Recording site)**
Median (APB)	S1: 8 cm proximal to active electrode, over median nerve at wrist S2: forearm at antecubital fossa	Active: Thenar eminence, midpoint of wrist crease to 1st MCP joint
Median sensory	Over median nerve at the wrist	2nd digit of hand, active at prox phalanx, reference 2 cm distal
Ulnar (ADM)	S1: 8 cm proximal to active electrode, over ulnar nerve at wrist. S2: Below elbow. S3: Above elbow	Active: Over abductor digiti minimi on hypothenar eminence
Ulnar sensory	Over the ulnar nerve at wrist	5th digit of hand, active at prox phalanx, reference 2cm distal
Radial (EDC)	S1: Antecubital fossa lateral to biceps tendon; S2: Proximal to the spiral groove	Active: Over extensor digitorum communis, distal to elbow
Radial sensory	Over radial nerve at the wrist	Distal ¼ point of forearm
Peroneal (EDB)	S1: Ankle over peroneal nerve; S2: Below fibular head; S3: Popliteal fossa	Active: Over extensor digitorum brevis on dorsum of foot
Tibial (AH)	Ankle and popliteal fossa	Active: Over abductor hallucis on medial foot
Sural	Mid-calf	Bar electrode posterior to lateral malleolus
Superficial peroneal	Mid-lateral leg	Bar electrode anterior ankle

Muscle abbreviations: APB, abductor pollicis brevis; ADM, abductor digiti minimi; EDC, extensor digitorum communis; EDB, extensor digitorum brevis; AH, abductor hallucis.

For data collection, the prevalence of positive Tinel signs at each anatomical site will be recorded for both groups, and the total number of sites per individual with positive Tinel sign will also be analyzed. The prevalence of positive Tinel signs in the hEDS group will be compared by statistical analysis to the control group using chi-square tests for categorical data. Logistic regression models will be employed to adjust for potential confounders such as age, gender, and occupation.

## Discussion

### Principal findings

This study is expected to demonstrate that individuals with hEDS exhibit a significantly higher prevalence of positive Tinel signs across multiple anatomical sites compared to controls. In addition, individuals with hEDS may display positive Tinel signs at atypical locations not commonly associated with entrapment syndromes in the general population. Subgroup analyses may also identify a correlation between the degree of joint hypermobility and features of small fiber neuropathy, which may or may not coincide with a positive Tinel sign.

### Clinical implications

If confirmed, these findings would offer a new perspective on the neuropathic symptoms frequently reported in individuals with hEDS (14). A higher prevalence of Tinel positivity may reflect a greater susceptibility to peripheral nerve irritation or compression due to underlying connective tissue fragility. Clinically, this underscores the importance of thorough and systematic peripheral nerve assessments in hEDS populations. Early recognition of nerve entrapment syndromes could lead to earlier intervention and potentially mitigate the progression of chronic neuropathic pain. These results may also inform the development of more targeted management strategies for neuropathic symptoms in this group.

### Strengths and limitations

A key strength of this study is its systematic approach to evaluating Tinel's sign across multiple anatomical sites in a clinically relevant population. The anatomical mapping of nerve irritation may yield novel insights into the distribution and pathophysiological patterns of nerve involvement in hEDS.

However, several limitations should be noted. Tinel's sign is a subjective clinical test with potential for inter-examiner variability. Its presence may be influenced by patient sensitivity, examiner technique, and lacks specificity for underlying pathology. Additionally, as a cross-sectional study, causal relationships between hypermobility, nerve irritation, and neuropathic symptoms cannot be established. The absence of confirmatory diagnostic tools such as nerve conduction studies, skin biopsies, or quantitative sensory testing may also limit the interpretability of findings. Future studies incorporating objective assessments of nerve function and longitudinal follow-up will be critical to validating and expanding upon these results.

### Future directions

To build on these findings, future research should incorporate objective neurophysiological and histopathological assessments, such as nerve conduction studies, corneal confocal microscopy, or intraepidermal nerve fiber density analysis. Longitudinal studies may also help to determine whether the presence of Tinel signs in hEDS is predictive of worsening neuropathic symptoms or long-term nerve dysfunction. Ultimately, a better understanding of peripheral nerve involvement in hEDS could contribute to earlier diagnosis, more effective interventions, and improved quality of life for affected individuals.

## Conclusion

Testing the hypothesis that hEDS individuals will have a higher prevalence of positive Tinel sign could provide insight into the neuropathic manifestations of the disorder, potentially guiding future diagnostic and treatment strategies. Further research is required to explore the clinical significance of this relationship and how it might influence patient care for hEDS.

## Data Availability

The original contributions presented in the study are included in the article/supplementary material, further inquiries can be directed to the corresponding author.

## References

[1] Malfait F Francomano C Byers P Belmont J Berglund B Black J. The 2017 international classification of the Ehlers-Danlos syndromes. Am J Med Genet C Semin Med Genet. (2017) 17:8–26. 10.1002/ajmg.c.31552 28306229

[2] Blackburn PR Xu Z Tumelty KE Zhao RW Monis WJ Harris KG. Bi-allelic alterations in Aebp1 lead to defective collagen assembly and connective tissue structure resulting in a variant of Ehlers-Danlos syndrome. Am J Hum Genet. (2018) 102:696–705. 10.1016/j.ajhg.2018.02.018 29606302 PMC5985336

[3] Syx D De Wandele I Rombaut L Malfait F. Hypermobility, the Ehlers-Danlos syndromes and chronic pain. Clin Exp Rheumatol. (2017) 107:116–22. 28967365

[4] Tinkle B Castori M Berglund B Cohen H Grahame R Kazkaz H. Hypermobile Ehlers-Danlos syndrome (a.k.a. Ehlers-Danlos syndrome type III and Ehlers-Danlos syndrome hypermobility type): clinical description and natural history. Am J Med Genet C Semin Med Genet. (2017) 175:48–69. 10.1002/ajmg.c.31538 28145611

[5] Davis EN Chung KC. The Tinel sign: a historical perspective. Plast Reconstr Surg. (2004) 114:494–9. 10.1097/01.PRS.0000132675.12289.78 15277821

[6] Beale S Duraku LS McGhee CCG van der Oest M Rotem G Power DM. SCOPING: a pilot study exploring the role of a series of clinical observational parameters as indicators of nerve regeneration [published correction appears in Plast Reconstr Surg Glob Open. (2024) 12:e6454. Plast Reconstr Surg Glob Open. (2024) 12:e6111. 10.1097/GOX.0000000000006111 39220753 PMC11365670

[7] Galan E Kousseff BG. Peripheral neuropathy in Ehlers-Danlos syndrome. Pediatr Neurol. (1995) 12:242–5. 10.1016/0887-8994(95)00003-X 7619192

[8] Fernandez A Aubry-Rozier B Vautey M Berna C Suter MR. Small fiber neuropathy in hypermobile Ehlers Danlos syndrome/hypermobility spectrum disorder. J Intern Med. (2022) 292:957–60. 10.1111/joim.13539 35781355 PMC9796626

[9] Igharo D Thiel JC Rolke R Akkaya M Weis J Katona I. Skin biopsy reveals generalized small fibre neuropathy in hypermobile Ehlers-Danlos syndromes. Eur J Neurol. (2023) 30:719–28. 10.1111/ene.15649 36437696

[10] Ishizuka K Uehara T Yokokawa D Noda K Ikusaka M. Hoffmann-Tinel sign and entrapment neuropathy. QJM. (2021) 114:45–6. 10.1093/qjmed/hcaa156 32369602

[11] Alfonso MI Dzwierzynski W. Hoffman-Tinel sign: the realities. Phys Med Rehabil Clin N Am. (1998) 9:721–36. 10.1016/S1047-9651(18)30229-8 9894091

[12] Feldman EL Stevens MJ Thomas PK Brown MB Canal N Greene DA. Michigan Neuropathy Screening Instrument (MNSI). Ann Arbor, MI: University of Michigan Health (1994).

[13] Buschbacher RM Prahlow ND. Manual of Nerve Conduction Studies, 2nd Edn. New York, NY: Demos Medical Publishing (2006).

[14] Nose M. A polygene network model for the complex pathological phenotypes of collagen disease. Pathol Int. (2011) 61:619–29. 10.1111/j.1440-1827.2011.02725.x 22029672

